# Understanding and Controlling Mode Hybridization in Multicavity Optical Resonators Using Quantum Theory and the Surface Forces Apparatus

**DOI:** 10.1021/acsphotonics.1c01055

**Published:** 2021-11-15

**Authors:** Bruno Zappone, Vincenzo Caligiuri, Aniket Patra, Roman Krahne, Antonio De Luca

**Affiliations:** † Consiglio Nazionale delle Ricerche − Istituto di Nanotecnologia (CNR-Nanotec), via P. Bucci 33/C, 87036 Rende, CS, Italy; ‡ Università della Calabria − Dipartimento di Fisica, via P. Bucci 31/C, 87036 Rende, CS, Italy; § Istituto Italiano di Tecnologia (IIT) − Optoelectronics Research Line, via Morego 30, 16163 Genova, Italy

**Keywords:** optical cavity, surface forces apparatus, quantum analogy, metal-dielectric multilayer, epsilon-near-zero modes

## Abstract

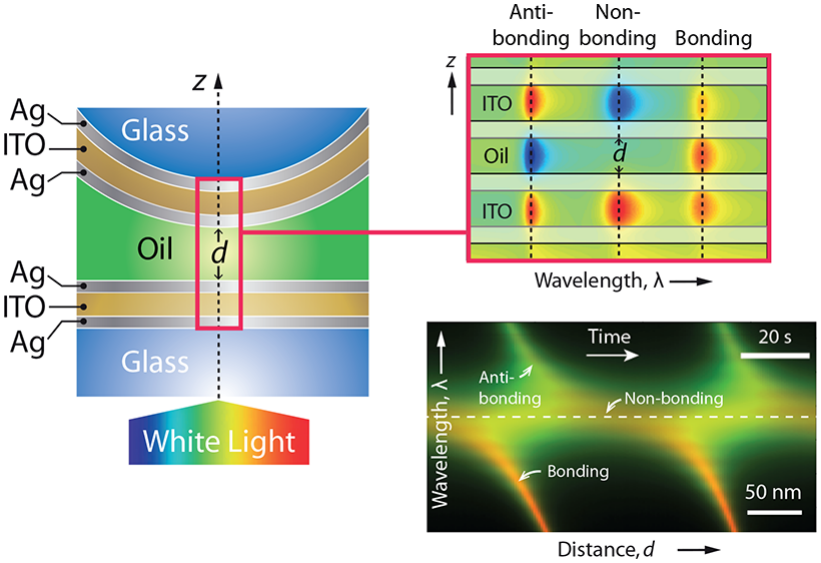

Optical fields in metal-dielectric multilayers display typical features of quantum systems, such as energy level quantization and avoided crossing, underpinned by an isomorphism between the Helmholtz and Schrödinger wave equations. This article builds on the fundamental concepts and methods of quantum theory to facilitate the understanding and design of multicavity resonators. It also introduces the surface forces apparatus (SFA) as a powerful tool for rapid, continuous, and extensive characterization of mode dispersion and hybridization. Instead of fabricating many different resonators, two equal metal-dielectric-metal microcavities were created on glass lenses and displaced relative to each other in a transparent silicone oil using the SFA. The fluid thickness was controlled in real time with nanometer accuracy from more than 50 μm to less than 20 nm, reaching mechanical contact between the outer cavities in a few minutes. The fluid gap acted as a third microcavity providing optical coupling and producing a complex pattern of resonance splitting as a function of the variable thickness. An optical wave in this symmetric three-cavity resonator emulated a quantum particle with nonzero mass in a potential comprising three square wells. Interference between the wells produced a 3-fold splitting of degenerate energy levels due to hybridization. The experimental results could be explained using the standard methods and formalism of quantum mechanics, including symmetry operators and the variational method. Notably, the interaction between square wells produced bonding, antibonding, and nonbonding states that are analogous to hybridized molecular orbitals and are relevant to the design of “epsilon-near-zero” devices with vanishing dielectric permittivity.

Analogies between optics and quantum mechanics date back to the foundation of quantum theory and continue to stimulate a fruitful exchange of ideas between these fields.^1,2^ For instance, non-Hermitian systems with parity-time symmetry^3,4^ and spin–orbit coupling in complex electronic structures^5^ are actively investigated with the help of photonic emulators providing “synthetic” Hamiltonians. Quantum theory also underpins the study of bound states in the continuum (BIC),^6−8^ which can be found in photonic structures such as photonic crystals^9−12^ and double-bend waveguides.^12^ It has long been known that the Helmholtz equation for optical waves in transparent dielectric materials is isomorphic to the steady-state Schrödinger equation for quantum wave functions.^1,13^ An electromagnetic field with time dependence e^–*i*ω*t*^ in a uniform isotropic material satisfies the Helmholtz wave equation ∇^2^ψ + ε(ω/*c*)^2^ψ = 0, where ψ is a component of the electric or magnetic field, ε is the dielectric permittivity, and *c* is the speed of light. Although the permittivity generally is a complex number, it can be approximated as a positive real number ε *= n*_D_^2^ > 0 in a nonabsorbing transparent dielectric at optical frequencies, where *n*_D_ is the refractive index. On the other hand, the imaginary part of ε is much larger than the real part in metals such as Ag and Mg, so that the permittivity can be approximated as a negative real number ε *=* κ_M_^2^ < 0, where κ_M_ is the extinction coefficient.^14^ Because the quantity ε(ω/*c*)^2^ is always close to a real number in a metal-dielectric structure, the Helmholtz equation is formally equivalent to the Schrödinger equation ∇^2^ψ + (2*m*/*ℏ*^2^)( – *V*)ψ = 0, describing a hypothetical quantum particle with mass *m* and energy  in a potential *V*, where ε(ω/*c*)^2^ plays the role of (2*m*/*ℏ*^2^)( – *V*).

A transparent dielectric corresponds to a region of space where  is larger than *V*, namely  = *V* + (ε/2*m*)(*ℏ*ω/*c*)^2^ with ε > 0, whereas a nonabsorbing metal with ε < 0 corresponds to  < *V* (Figure 1). A piece-wise uniform synthetic potential *V* can be obtained simply by joining materials with different real permittivities. The potential undergoes a step-like variation at the interface between a transparent dielectric and a nonadsorbing metal, increasing by the quantity *U* = (*ℏ*ω/*c*)^2^(*n*_D_^2^ + κ_M_^2^)/2*m* from the dielectric into the metal (Figure 1). Through the Helmholtz–Schrödinger isomorphism, quantum mechanics provides a powerful theoretical toolbox to understand the optics of metal-dielectric structures. Vice versa, typical quantum features such as energy level quantization and hybridization can be engineered, controlled, and studied more conveniently in metal-dielectric structures than in genuine quantum systems.

**Figure 1:**
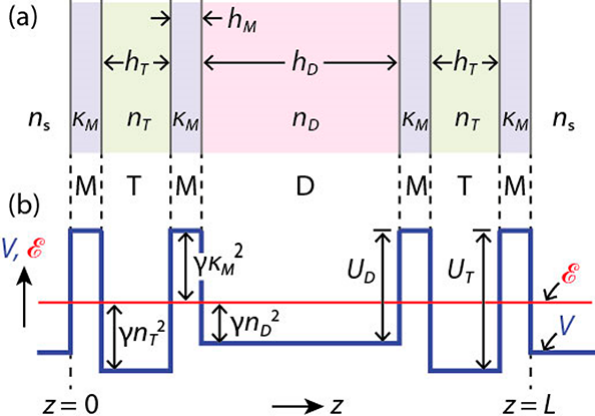
(a) Planar multilayer comprising metal (M) and transparent dielectric (T) layers with fixed thickness *h*_M_ and *h*_T_, respectively, and a transparent dielectric film (D) with variable thickness *h*_D_. The outer MTM cavities and central MDM cavity form a symmetric three-cavity resonator (MTMDMTM). *n*_T_, *n*_D_, and *n*_s_ are the refractive index of the T and D layers, and of the surrounding dielectric medium, respectively, whereas κ_M_ is the metal extinction coefficient. (b) Synthetic quantum potential *V* comprising three square wells with depths *U*_T_ or *U*_D_, corresponding to the T and D layers.  is the energy of the quantum particle and γ = (ℏω/*c*)^2^/2*m*.

From both the experimental and conceptual points of view, the simplest metal-dielectric optical device is the planar cavity obtained by sandwiching a layer of solid dielectric material (T) between two partially reflecting metal layers (M). Such MTM cavity, also known as Fabry–Perot etalon,^15,16^ can be obtained by sequential vapor deposition or sputtering of the M and T materials on glass.^17^ Multiple-beam interference between light waves bouncing on the metal mirrors modulates the cavity’s optical transmittance, producing a discrete sequence of sharp resonance peaks at wavelengths λ_*p*_, where *p* is the chromatic order of the resonance mode (Figure 2, red curves).

**Figure 2:**
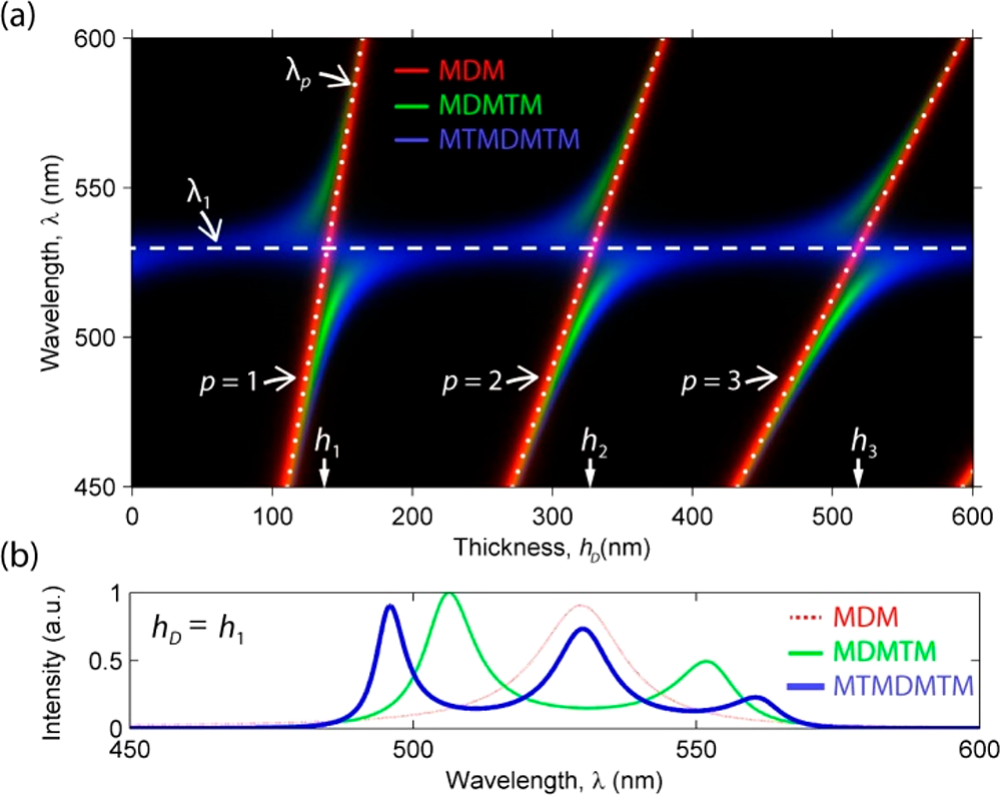
(a) Transmittance under normal incidence calculated with the transfer matrix method for a single cavity (MDM, red), asymmetric two-cavity resonator (MDMTM, green), and symmetric three-cavity resonator (MTMDMTM, blue). M, T, and D indicate respectively a metal (Ag), rigid transparent material (ITO), and deformable transparent dielectric (silicone oil). The transmittance is shown as a function of the thickness *h*_D_ of the D layer and wavelength λ. Resonances correspond to local intensity maxima. The M and T layers had thickness *h*_M_ = 40 nm and *h*_T_ = 85 nm, respectively. The T and D layers had refractive indices of *n*_T_ = 1.9 and *n*_D_ = 1.4, respectively. White dots indicate the resonant wavelengths of the MDM cavity calculated as λ_*p*_ ≈ 2*nh*_D_*/*p* where *p* is the resonance order and *h*_D_* = *h*_D_ + 50 nm is an effective thickness. λ_1_ is the first resonant wavelength of a single MTM cavity. The wavelengths λ_*p*_ of a single MDM cavity cross λ_1_ at thicknesses *h*_D_ = *h*_1_, *h*_2_, ..., *h*_*p*_. (b) Transmitted spectra for the thickness *h*_D_ = *h*_1_ showing a 2-fold wavelength splitting (green curve) or 3-fold splitting (thick blue curve) for the MDMTM and MTMDMTM resonator, respectively, compared to the single resonance (dotted red curve) of the MDM cavity.

The transmittance can be precisely calculated using standard methods such as transfer matrix multiplication (see Supporting Information, SI, for details on the method).^15,18^ Cavity resonators are widely used in interferometry,^15^ lasers,^19^ spectroscopy and molecular sensing,^20^ color filters, superabsorbers,^21^ thin-film studies^22^ and surface force measurements.^23^ Moreover, a MTM cavity can be described as a single homogeneous layer with an effective dielectric response such that the real part of the permittivity crosses zero at resonance, while the imaginary part becomes very small.^14^ Such “epsilon-near-zero” (ENZ) permittivity is associated with many intriguing phenomena, including nonlinearity enhancement,^24^ negative refraction,^25,26^ ultrafast optical switching,^27^ adiabatic frequency shifting,^28^ intraband optical conductivity,^29^ phase singularity,^30^ and appearance of Casimir forces.^31^ Compared to natural ENZ materials such as Ag and indium–tin-oxide (ITO), waveguides^32^ and hyperbolic metamaterials,^33^ metal-dielectric cavity resonators provide a simple and flexible design of ENZ modes with low losses in the visible spectrum, which can be used to engineer strong light-matter coupling.^34^

Multicavity planar resonators can be obtained by stacking two or more metal-dielectric cavities with shared metal layers. In an asymmetric two-cavity resonator (MTMDM) with a deformable dielectric (D) layer, the coupling between resonance modes of the MTM cavity (e.g., Figure 2a, dashed horizontal line with wavelength λ_1_) and MDM cavity (Figure 2a, dotted lines with wavelengths λ_*p*_) leads to avoided crossings (Figure 2a, green curves at thicknesses *h*_D_ = *h*_1_, *h*_2_, ...) and splitting of resonance wavelengths (Figure 2b, green curve). These effects are due to the hybridization of single-cavity modes analogous to the creation of delocalized molecular orbitals from single-atom orbitals.^34^ The analogy can be extended to periodic cavity resonators, showing ENZ photonic bands similar to electron bands in solid-state crystals.^35^ Yet, a theoretical and mathematical framework is needed to calculate the optical coupling strength and explain how the complex dispersion of a multicavity resonator originates from the simple responses of individual cavities.

This article introduces the surface forces apparatus (SFA) as a powerful tool to study the modal dispersion of multicavity resonators. Cavity thickness was varied rapidly, continuously, extensively, and with nanometer accuracy. This allowed measuring the dispersion as a function of cavity thickness (as in Figure 2a), avoiding the costly and time-consuming experimental task of fabricating multiple resonators with different thicknesses. We validated this approach for symmetric three-cavity resonators (MTMDMTM) with a deformable dielectric layer (D), exhibiting a pattern of 3-fold wavelength splitting (Figure 2, blue color). On the basis of the Helmholtz–Schrödinger isomorphism with a quantum potential comprising three interfering square wells, our study shows that the 3-fold splitting reflects the formation of a “non-bonding” hybrid mode in addition to the “bonding” and “anti-bonding” modes of a two-cavity resonator. Interestingly, the nonbonding mode is insensitive to variations of optical thickness in the central D layer.

We anticipate that the SFA can be used to study virtually any planar optical multilayer as a function of the thickness of one or more layers, for example, to characterize the coupling of epsilon-near-zero modes with excitons embedded in a fluid or create planar optical metamaterials with tunable optical response. Our findings also have implications in the design and interpretation of SFA experiments on the electrochemistry of surfaces and the electrification of nanoscale fluid films, typically involving multicavity metal-dielectric resonators.^36−39^

## Results

### Transmittance of a Single Cavity

**Figure 3:**
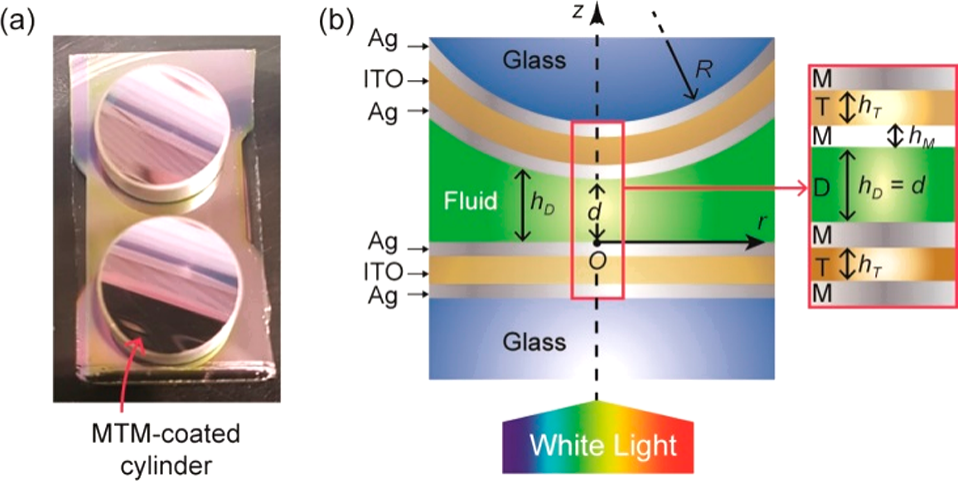
(a) MTM microcavities fabricated on the SFA cylindrical lenses having 1 cm diameter and radius of curvature *R* = 2 cm. The M and T layers were made of Ag and ITO, respectively. (b) Schematic of the SFA setup with two MTM-coated cylinders facing each other in silicone oil (D) at distance *d* apart. The cylinder axes are crossed at 90°, ensuring a single surface contact point (*r* = 0, dashed vertical line) around which the surface separation distance *h*_D_ approximates a sphere-plane geometry (eq 1). The inset shows the planar MTMDMTM resonator obtained at the contact point.

Rigid MTM microcavities were fabricated by sputtering deposition of Ag and ITO on glass with target thickness *h*_M_ = 40 nm and *h*_T_ = 80 nm, respectively. The cavities were produced both on planar glass slides and on the cylindrical lenses used in SFA experiments, which have a diameter of 1 cm and curvature radius *R* = 2 cm (Figure 3a). The transmittance of planar cavities was measured by ellipsometry and showed a peak at wavelength λ_1_ = 520 nm, with full width at half-maximum of about 40 nm.

The transmittance of a single cavity (MDM) with variable thickness *h*_D_ was calculated as a function of the wavelength λ using the transfer matrix method (Figure 2, red curves).^15^ For ideal perfectly reflecting metal layers with zero electric resistivity, the resonant modes of a MDM cavity are confined within the D layer. Multiple-beam interference produces a set of “quantized” wavelengths λ*_p_* = 2*n*_D_*h*_D_/*p*, corresponding to resonant modes with different order *p* = 1, 2, 3, ..., where *n*_D_ is the refractive index of the D layer.^15^ In real metals with finite conductivity, the field extends into the M layer over a distance δ ≈ λ/2πκ known as skin depth, where κ is the metal extinction coefficient, producing a red-shift of the resonance wavelengths. For Ag, κ ≈ 3 is almost constant and δ ≈ 27 nm varies less than 10% across the optical spectrum (450–600 nm wavelengths).

Figure 2 shows that the resonance wavelengths of a single cavity vary as a function of the thickness *h*_D_ according to an approximately linear relation: λ*_p_* = 2*n*_D_*h*_D_*/*p*, where *h*_D_*> *h*_D_ is an effective thickness including the skin depth.^22^ In particular, the 520 nm peak of planar rigid MTM cavities corresponded to the first resonant mode with λ_1_ ≈ 2*n*_T_*h*_T_*, where *n*_T_ = 1.8 and *h*_T_* = 144 nm are the ITO refractive index and effective thickness, respectively. Note that *h*_T_* is close to *h*_T_ + 2δ ≈ 134 nm, where *h*_T_ is the target ITO thickness.

### Dispersion of a Three-Cavity Resonator

The SFA was originally developed to measure surface interactions in fluid films and soft materials with nanoscale thickness.^40,41^ The material is confined between two cylindrical solid surfaces with a radius of curvature *R* of a few cm, which are mounted in a sealed enclosure at a distance *d* apart with their cylinder axes crossed at 90°, ensuring a single contact position (Figure 3b). The surface separation distance is
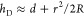
1approximating a sphere-plane geometry at small lateral distances *r* ≪ *R* from the contact position (*r* = 0). The top surface is fixed to a rigid mount, whereas the bottom surface is attached to the free end of a two-spring cantilever. The distance *d* can be varied with nanoscale accuracy by displacing the fixed cantilever end with precision linear actuators. In this work, two MTM-coated cylindrical lenses (Figure 3a) were approached at a distance *d* in silicone oil (Figure 3b). The fluid gap (D) between the two rigid cavities acted as a third deformable microcavity (MDM), coupling the outer rigid cavities in a symmetric three-cavity resonator (MTMDMTM) with variable thickness of the D layer (Figure 1, Figure 2, blue color, and Figure 3b, inset). The optical transmittance was measured under normal incidence at the surface contact point (*r* = 0, Figure 3b) while varying the surface distance *d* from more than 50 μm to less than 20 nm, reaching direct mechanical contact between the MTM cavities, in a single sweep taking less than 10 min. This amounted to varying the thickness *d* of the fluid film in the central cavity (MDM) while keeping the outer cavities (MTM) unchanged.

Transfer matrix calculations show that the avoided crossing between resonances of the central fluid cavity (MDM) and outer rigid cavities (MTM) leads to a 3-fold splitting of the resonant wavelength (Figure 2, blue curves). The calculations also show that the central wavelength of the triplet is almost constant as a function of the fluid film thickness *h*_D_ and close to the first-mode wavelength λ_1_ of the outer cavities. Moreover, the resonance of the three-cavity resonator (Figure 2, blue curves) overlaps with that of a single MDM cavity (Figure 2, red curves and white dots) outside the avoided crossing region, i.e., when *h*_D_ is far from *h*_*p*_ or, equivalently, when λ*_p_* is far from λ_1_.

**Figure 4:**
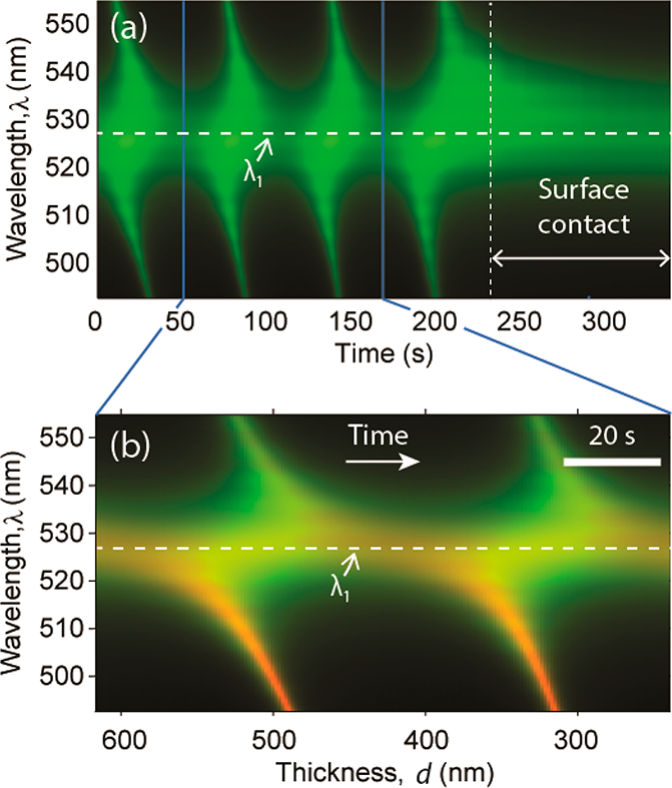
(a) Experimental SFA spectrogram showing the intensity transmitted by a symmetric three-cavity resonator (MTMDMTM) at the contact position (*r* = 0 in Figure 3b) as a function of time and wavelength λ during surface approach with cantilever speed *u* = 3.3 nm/s. In the surface contact region, a mechanical force slowed the surface motion and time evolution of the transmitted spectrum. λ_1_ is the first-order wavelength of the outer MTM cavities. (b) Overlay of the experimental spectrogram (green) and calculated transmittance (red) showing the noncontact region where the surface distance *d* varied uniformly with speed *u*. Overlapping intensities appear orange. In the transfer matrix calculation, the M and T layers had thickness *h*_M_ = 37 nm and *h*_T_ = 84 nm, respectively, and the D layer had refractive index *n*_D_ = 1.41.

These features were reproduced in SFA experiments (Figure 4a). The intensity *I* transmitted at the contact point (*r* = 0, Figure 3b) was measured as a function of the wavelength λ and time while displacing the fixed end of the cantilever at a constant speed *u* = 3.3 nm/s. At large distances *d*, surface interactions were negligible.

The cantilever was not deflected and the bottom surface was displaced at the same speed as the cantilever, i.e., ∂*d*/∂*t* = *u* (Figure 4a, left side). Therefore, the evolution of the transmission spectrum as a function of time corresponded to a linear variation of the fluid thickness *d* with time. When the surfaces reached contact, a repulsive mechanical force deflected the cantilever. The bottom surface moved at speed ∂*d*/∂*t* < *u*, and therefore, the time evolution of the spectrum slowed (Figure 4a, right side). The experimental *I*(*d*, λ) spectrograms showed local transmission peaks arranged in *S*-shaped fringes that were connected by a thickness-independent horizontal band, centered at wavelength λ_1_ = 520 nm (Figure 4a). These findings could be reproduced using transfer matrix calculations (Figure 4b), displaying the same features as in Figure 2. Namely, the *S*-shaped fringes appeared at fluid thicknesses close to *h*_*p*_, where a 3-fold resonance splitting was expected, and the horizontal band corresponded to the central wavelength of the triplet, close to the resonant wavelength λ_1_ of the MTM cavities.

**Figure 5:**
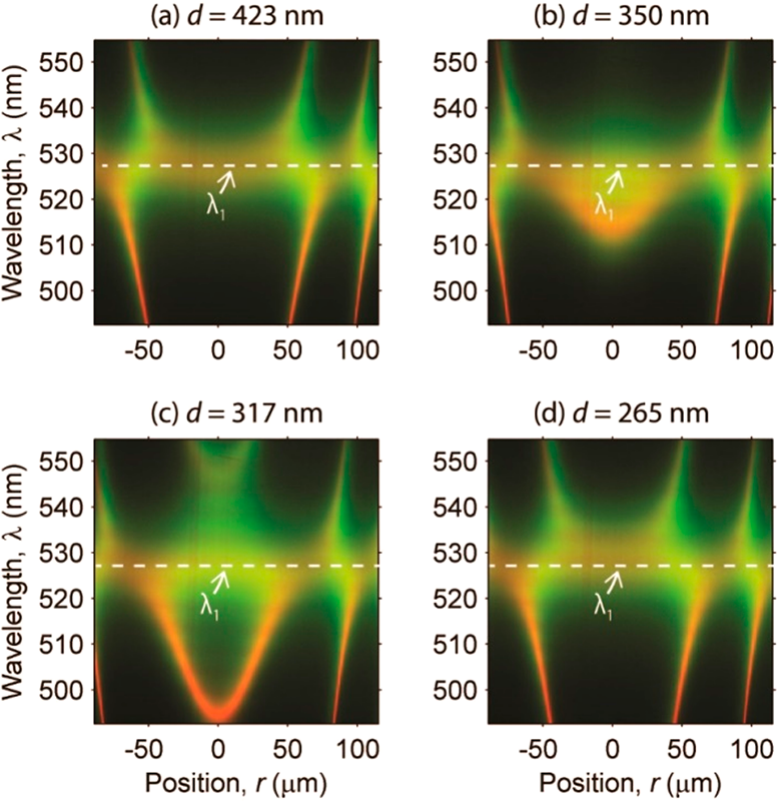
Experimental (green) and simulated (red) spectrographs showing the intensity transmitted by the MTMDMTM resonator as a function of the wavelength λ and lateral position *r* for different surface separation distances *d*. The surfaces were continuously approached going from (a) to (d). λ_1_ is the first-order wavelength of the outer MTM cavities. The transfer matrix calculation parameters are the same as in Figure 4b.

The SFA setup includes an imaging spectrograph that resolves the intensity *I* as a function of the wavelength λ and lateral distance *r* from the contact position (Figure 5).^40^ For a fixed distance *d*, the resonant wavelengths varied as a function of *r* in a way that reflected the dependence of *r* on *h*_D_. Namely, *h*_D_ increased parabolically with *r* (eq 1) and the resonant wavelength increased almost linear with *h*_D_ far from λ_1_ (Figure 2). In this region, therefore, the resonance fringes λ(*r*) had a parabolic shape. A single *I*(*r*, λ) spectrograms could be obtained in less than a second and covered a range of thickness *h*_D_ of about 300 nm, from *d* to *d* + *r*_max_^2^/2*R*, where *r*_max_ ≈ 100 μm is half the field of view of the spectrograph.

## Discussion

### Quantum Theory in a Multiwell Potential

Consider a multicavity resonator with total thickness *L* and layer normal *z*, so that *z* = 0 and *z* = *L* correspond to the resonator’s entrance and exit, respectively (Figure 1b). Under the condition of normal incidence used in SFA experiments, the Helmholtz–Schrödinger isomorphism applies to any transverse component ψ of the electric or magnetic field in any layer of the resonator (under oblique incidence, the isomorphism is valid only for transverse-electric plane waves, as shown in SI).

The resonator’s transmittance is proportional to the square modulus |ψ|^2^ for *z* > *L*. This is equivalent to the probability |ψ(*z* > *L*)|^2^ of finding the quantum particle past the potential *V* created by the metal (M) and dielectric (T, D) layers (Figure 1). Electromagnetic field penetration in the M layers with skin depth δ = *c*/(2κω) is analogous to quantum tunneling in a potential barrier, with a tunneling distance equal δ = [2*m*(*U* – )/*ℏ*^2^]^−1/2^. Adopting the quantum formalism, we denote with |ψ⟩ an electromagnetic mode with wave function ψ(*z*) and introduce the complex scalar product ⟨ψ_1_|ψ_2_⟩ = ∫ψ_2_*ψ_1_ d*z*. The Helmholtz–Schrödinger isomorphism maps resonant modes into quantum eigenstates |ψ⟩ of a synthetic Hamiltonian with wave function representation:
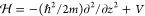
2Indeed, the resonator’s transmittance is different from zero at a frequency ω only if a nonzero solution ψ exists for the Helmholtz equation at that frequency. The same wave function is a solution of the Schrödinger equation , where  is the energy. Note that , *V*, and  – *V* depend on the frequency ω, and the energy zero is arbitrary.

Because *V* >  in the metal, whereas *V* <  in the dielectric materials, the synthetic potential *V* in eq 2 comprises a series of square wells corresponding to the T and D layers (Figure 1b). If the outer metal layers of the resonator are considered infinitely thick and the energy zero is in the metal, the synthetic potential is *V* = Σ_α_*V*_α_, where *V*_α_ is the square well potential corresponding to a single cavity. Namely, *V*_α_ = *U* = −(*ℏ*^2^*k*^2^/2*m*)(*n*_D_^2^ + κ_M_^2^) in the well and *V*_α_ = 0 elsewhere.^14^

Solutions to the wave equation for a single square well with potential *V*_α_ can be found in various textbooks on quantum mechanics.^42−44^ Because in our case  ≤ 0 (Figure 1b), the energy eigenstates |α_*p*_⟩ of a single cavity are bound states, i.e., the quantum particle is localized mainly in the well (a method for creating free particle states such that  > max(*V*) is outlined in SI). The eigenstate wave function ψ_α_ is real and has a defined wavevector modulus *nk*_*p*_ = *p*π/*h*_α_*, where *p* is the “quantum number” corresponding to the chromatic order, and *h*_α_* is an effective width. The mode of order *p* has a nondegenerate energy _α,*p*_ = (*ℏ*^2^/2*m*)(*nk*_*p*_)^2^. The potential *V*_α_ is invariant under the spatial inversion *z* → −*z* about the center of the well. Therefore, the single-cavity Hamiltonian,, commutes with the inversion operator and energy eigenstates |α_*p*_⟩ have a defined parity. Namely, eigenstates with odd or even order *p* are even or odd, respectively.^42−44^

Solutions of the Schrödinger equation for a double square well potential can be found in quantum physics textbooks.^45^ Multiple square wells appear in the study of stacked semiconductor quantum wells, chains of quantum dots, and Bose–Einstein condensates.^46^ Examples of multiple coupled resonators abound in mechanics, electronics, optics, and photonics and can be analyzed using general methods such as coupled-mode theory and coupled-oscillator models.^47−50^ In this work, we present a method of analysis based on a three-well potential that reproduces the experimental findings (Figures 4 and 5), establishes a clear connection with well-known concepts and methods of quantum mechanics, and allows direct calculation of the coupling coefficients and wavelength splitting.

A symmetric MTMDMTM resonator is analogous to a symmetric three-well synthetic potential (Figure 1b). In the absence of interference between wells, the energy eigenstates |α_*p*_⟩ = |*l*_*p*_⟩ and |α_*p*_⟩ = |*r*_*p*_⟩ of the left and right well, respectively, have equal order *p* and equal energy _*o*,*p*_, whereas the eigenstates |*c*_*q*_⟩ of the central well with order *q* have *a priori* different energies. For three noninterfering wells, the energy  is nondegenerate when it matches a level _*c*,*q*_ of the central well but none of the levels _*o*,*p*_ of the outer wells. A 2-fold degeneracy is found when  matches a level of the outer wells without matching any level _*c*,*q*_ of the central well. A 3-fold degeneracy is found when  = _*o*,*p*_ = _*c*,*q*_*.* When the refractive index is the same in all cavities, the 3-fold degeneracy occurs when the ratio *h*_D_*/*h*_T_* between the widths of central and outer cavities is a rational number, so that *q*/*h*_D_* = *p*/*h*_T_* for a suitable choice of integers *p* and *q* (Figure 1).

### Variational Method Analysis

In our experiments, the distance *h*_M_ between wells, that is the thickness of the metal layers, was comparable but larger than the tunneling length (skin depth) δ (Figure 1). Therefore, the wave function overlap between neighboring wells was small. To quantify the wavelength splitting and coupling strength, we use the Rayleigh–Ritz variational method, a classical tool to study the hybridization of atomic orbitals in molecular quantum theory.^36,41^ Namely, hybridized states are written as linear combination |ψ⟩ = Σ_α_*a*_α_|α_*p*_⟩ of the energy eigenstates |α_*p*_⟩ of isolated noninterfering wells. The coefficients *a*_α_ are obtained by applying the variational condition ∂/∂*a*_α_ = 0, where  is the average energy of |ψ⟩ and  is the total Hamiltonian of the interfering wells (eq 2).

**Figure 6:**
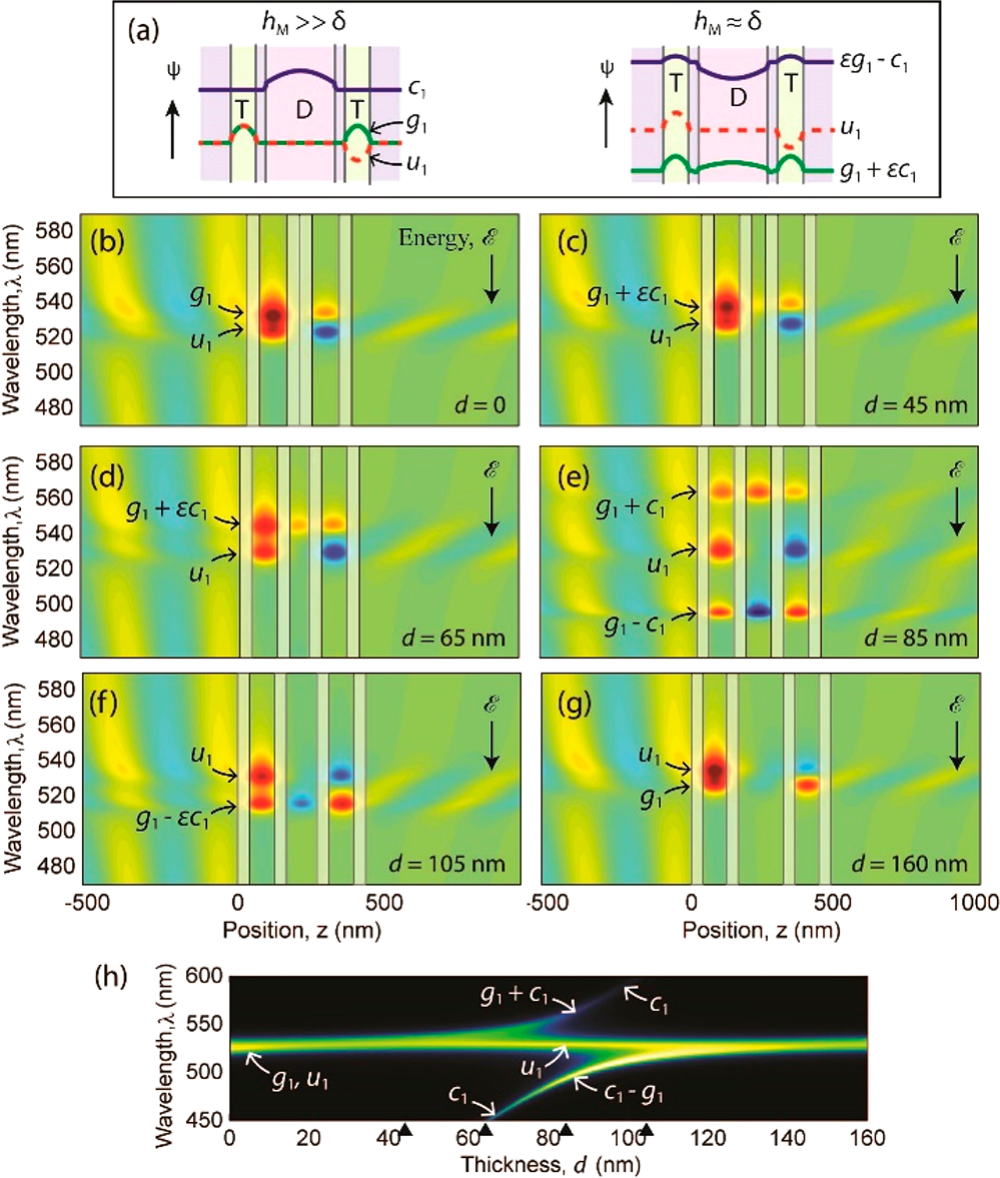
(a) First-order states of the central cavity (|*c*_1_⟩) and of the outer cavities (|*g*_1_⟩ and |*u*_1_⟩), shown to the left, produce the hybridized states shown to the right for a metal thickness *h*_M_ ≈ δ. ϵ is a positive hybridization coefficient. (b–g) Real part of the electric field calculated as a function of the position *z* along the layer normal and wavelength λ for various thicknesses *d* of the central fluid layer (D). The resonator was illuminated under normal incidence from the left side (*z* < 0). Shaded areas indicate the metal (M) layers. (h) Calculated transmittance. The transfer matrix calculation parameters are the same as in Figure 4b.

Because SFA spectrographs only captured the wavelength λ_1_ = 520 nm of the first-order states |*l*_1_⟩ and |*r*_1_⟩ for the outer wells (Figures 4 and 5), we only consider the hybridization of these states with the states |*c*_*q*_⟩ of the central well. Quantum perturbation theory shows that the hybridization is strongest for the |*c*_*q*_⟩ state with energy _*c*,*q*_ closest to the first-order energy _1*l*_ = _1*r*_ of the left and right wells.^42,51^ Therefore, trial functions for the variational method can be written as
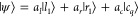
3These functions should be eigenstates of the symmetry operator *P* that inverts the *z-*axis about the midpoint of the central well (*z* = *L*/2 in Figure 1b). The operator *P* exchanges |*l*_1_⟩ and |*r*_1_⟩, that is *P*|*l*_1_⟩ = |*r*_1_⟩ and *P*|*r*_1_⟩ = |*l*_1_⟩, so that the combinations |*g*_1_⟩ = (|*l*_1_⟩ + |*r*_1_⟩)/√2 and |*u*_1_⟩ = (|*l*_1_⟩ – |*r*_1_⟩)/√2 are even and odd, respectively (Figure 6a, left side). In particular, the first-order even state |*c*_1_⟩ can be combined with |*g*_1_⟩ to create the even states:
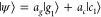
4with a suitable choice of the coefficients *a*_*g*_ and *a*_*c*_ (Figure 6a, right side). On the other hand, |*c*_1_⟩ cannot create a state with defined parity by mixing with |*u*_1_⟩, which therefore is the only odd hybrid state.

### Bonding, Antibonding, and Nonbonding Modes

To connect SFA experiments (Figures 4 and 5) and quantum theory, suppose to increase the width *h*_D_ of the central well/cavity. For *h*_D_ = 0, the coefficient *a*_*c*_ = 0 should be used in eq 3 and therefore the trial functions are |ψ⟩ = |*g*_1_⟩ and |ψ⟩ = |*u*_1_⟩. Since the left and right cavities are in direct contact (MTMMTM), they can couple to each other through the double metal layer at the center. This layer, however, acts as an effective double-width barrier to tunneling and hinders wave function overlap between the cavities. As shown in the SI, a negligible overlap leads to a negligible difference between the energies _1*g*_ and _1*u*_ of the states |*g*_1_⟩ and |*u*_1_⟩, respectively. This also entails that _1*g*_ and _1*u*_ are practically equal to the first-order energy _1*l*_ = _1*r*_ of noninterfering outer wells. The absence of energy splitting explains why transfer matrix calculations (Figure 2, blue curves) and SFA spectrograms (Figure 4) do not show any significant splitting of the first-order wavelength λ_1_ for a vanishing thickness *h*_D_ of the central cavity. The splitting and symmetry of the |*g*_1_⟩ and |*u*_1_⟩ states are highlighted by transfer matrix calculations in Figure 6b, showing the real part of the electric field. On the other hand, it has been shown that a thick central metal layer increases the quality factor of a two-cavity resonator, which behaves as a superabsorber.^34,52^ Moreover, the resonator shows ENZ permittivity at resonance.^34^

As the thickness *h*_D_ of the central well increases, the level _1*c*_ of the central-well state |*c*_1_⟩ approaches from above the level _1*g*_ ≈ _1*u*_ of the outer-well states |*g*_1_⟩ and |*u*_1_⟩. Because |*u*_1_⟩ does not mix with |*c*_1_⟩ and has negligible intensity within the inner cavity (Figure 6b–g), |*u*_1_⟩ is practically independent of the thickness *h*_D_ and refractive index *n*_D_ of the central cavity. This type of state is called “non-bonding” in molecular orbital theory^53^ and corresponds to the horizontal band with wavelength λ_1_ observed in the calculated (Figure 2) and experimental SFA spectrograms (Figures 4 and 5) for all thicknesses *h*_D_. On the other hand, the states |*g*_1_⟩ and |*c*_1_⟩ become increasingly hybridized as *h*_D_ increases and _1*c*_ approaches _1*g*_ ≈ _1*u*_. Applying the variational conditions to the even trial function of eq 4 we obtain
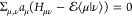
5where the indices μ and ν indicate |*g*_1_⟩ and |*c*_1_⟩, and  are the matrix elements of the total Hamiltonian  of the three interfering wells. Eq 5 admits two solutions with different energies  and coefficient ratios *a*_*c*_/*a*_*g*_. When the energy difference _1*c*_ – _1*g*_ is large, the ratio *a*_*c*_/*a*_*g*_ is either very large or very small, meaning that the hybridization is weak.^42,51^ The former case corresponds to a state close to |*c*_1_⟩, whereas the latter corresponds to a state close to |*g*_1_⟩ (indicated respectively as and ε|*g*_1_⟩ – |*c*_1_⟩ and |*g*_1_⟩ + ε|*c*_1_⟩ in Figure 6a, right side). As *h*_D_ and _1*c*_ – _1*g*_ decrease, the hybridization becomes stronger and the energy level of the |*c*_1_⟩-like state pushes the level of the |*g*_1_⟩-like state toward lower energies (compare Figures 2 and 4 with Figure 6c–f). This sort of repulsion demonstrates the von Neumann–Wigner level avoidance rule, according to which the levels of two hybridized states with the same symmetry cannot cross as a function of the single parameter *h*_D_.^51^

As *h*_D_ equals the thickness *h*_T_ of the left and right wells (Figure 1), and _1*c*_ reaches _1*g*_, the hybridization becomes strongest. As shown in the SI for three interfering wells having the same depth *U* and width *h*_D_, eq 5 leads to the two even hybrid states |±⟩ = (|*c*_1_⟩ ± |*g*_1_⟩)/√2 with different energies _±_ χ ≈ _1_ ± χ*U.* Here, χ is the overlap integral between |*c*_1_⟩ and |*g*_1_⟩ restricted to either the central well or the outer wells. Since χ*U* < 0, the |+⟩ state has energy _+_ < _1_ and “bonding” character, whereas the |−⟩ state has energy _–_ > _1_ and “anti-bonding” character. We stress that the variational method allows calculating *a priori* the off-diagonal matrix elements *H*_μν_ representing the coupling strength and, therefore, the splitting _*+*_ – _*–*_ (as shown in SI), in contrast with other methods where phenomenological coupling parameters must be determined *a posteriori*.^50^Figure 6e shows that the coupling produces a triplet state with the bonding and antibonding wavelength symmetrically spaced from the wavelength λ_1_ of the nonbonding state, as expected from simulations (Figure 2) and observed experimentally (Figure 4b). Note that coupling and interference between the wells completely removes the triple degeneracy of the energy level _*1c*_ = _1*u*_ = _1*g*_ expected for *h*_D_* = *h*_T_*.

When the level _1*u*_ = _1*g*_ is approached from above by the next level _2*c*_ of the central cavity, the odd state |*c*_2_⟩ is hybridized with |*u*_1_⟩ and pushes the perturbed level _*1u*_ to lower energy without perturbing the even state *|g*_1_⟩. The process repeats for higher-order modes of the central cavity. A video is provided as SI to show this process. Note that Figures 6(b–g) are single frames taken from the video.

In our experiments, the three-cavity resonator was surrounded by a dielectric medium with refractive index *n*_s_ (Figure 1 and 3b). Since the thickness of the outer metal layers was *h*_M_ ≈ δ, the optical waves were not completely confined within the resonator, but could penetrate from and leak into the surrounding medium via tunneling. Figure 1 shows that the optical transmittance of a single MTM cavity with Ag layer thickness *h*_M_ ≈ δ is maximum at essentially the same wavelengths λ*_q_* expected for a single square well. The difference with the *h*_M_ ≫ δ case is that the transmittance peaks of a single cavity have finite width and intensity, rather than having the shape of a Dirac delta function. Therefore, the three-cavity resonator shows essentially the same peak wavelengths (Figures 4 and 5) as if the outer metal layers of the resonator were infinitely thick.

## Conclusions

This article demonstrates that the SFA is a powerful tool to investigate the coupling of resonance modes in metal-dielectric multilayers. Mode dispersion can be measured rapidly, extensively, continuously, and accurately in a single sweep of cavity thickness using one resonator, eliminating the time and cost of fabricating many fixed-thickness resonators. The experimental dispersion curve of a symmetric three-cavity resonator showed a pattern of wavelength splitting as a function of the central cavity thickness that is qualitatively different from those of a single cavity and two-cavity resonator. To understand these findings, we established a framework of interpretation based on an isomorphism between the optical field in metal-dielectric multilayers and a quantum particle in a one-dimensional assembly of multiple square wells. The experimental findings could be explained by adopting the concepts, formalism, and methods of quantum theory, notably symmetry operators and the Rayleigh–Ritz variational method.

We anticipate that the SFA can be used to capture at a glance the optical response of a wide variety of resonators beyond the specific case considered in this article. For instance, a symmetric two-cavity resonator (MDMDM) can be created using a deformable dielectric material D for both cavities, e.g., a soft transparent elastomer such as poly(dimethyl-siloxane) (PDMS). The thickness of the cavities will decrease symmetrically in response to a mechanical load applied to the outer metal layers. Moreover, the SFA can be used to investigate the coupling of cavity modes with optical excitons, which is difficult to achieve using fixed-thickness resonators.

Our findings also have implications in the design and interpretation of SFA experiments on the electrochemistry of surfaces and the application of electric fields to nanoscale fluid films. In the standard SFA setup, the fluid (D) is confined between two molecularly smooth sheets of transparent mica (T), coated on the outside with partially reflecting Ag layers (M).^40,41^ The fluid thickness can be determined as a function of the resonance wavelength using an analytic expression valid for a single cavity (MT_1_DT_2_M) with a composite dielectric layer (T_1_DT_2_).^22^ To apply an electric field, metal electrodes such as gold layers can be introduced at the fluid-mica interfaces.^36−38^ However, these layers act as additional mirrors and create a multicavity resonator, producing additional features that should be analyzed using more advanced theoretical tools such as quantum theory.

## Materials and Methods

### MTM Fabrication and Preliminary Characterization

The MTM cavities were created by depositing layers of Ag and transparent indium–tin-oxide (ITO), respectively with target thickness 30 and 80 nm, on cylindrical glass lenses with radius *R* = 2 cm. The lenses had 1 cm diameter, 4 mm thickness, 60/40 scratch/dig surface quality, centration wedge angle <5 arcmin, and irregularity (interferometer fringes) λ/2 at a wavelength of 630 nm. Ag was chosen for its large extinction coefficient κ > 1 ≫ *n*, ensuring a high reflectivity and an approximately real negative permittivity in the metal layers.^14^ ITO was chosen for its transparency (*n* > 1 ≫ κ) and straightforward deposition via DC sputtering. For both Ag and ITO layers, the growth rate was 0.16 nm/s, the presputtering pressure was 3 × 10^–5^ mbar, and the sputtering pressure was 4.6 × 10^–2^ mbar. The power was 20 W for Ag and 40 W for ITO. The thickness and refractive index of Ag and ITO were determined in planar glass slides by ellipsometry, including extinction coefficients.

### SFA Measurements

The SFA Mark III by Surforce LLC, USA was used in the experiments.^40^ The elastic constant of the cantilever supporting the bottom surface was 900 N/m (Figure 2b). The central cavity between the surface-supported MTM cavities was initially filled with nitrogen, but mechanical vibrations blurred the transmitted spectra (not shown). A 40 μL droplet of silicone oil (PDMS from VWR/Prolabo #84543.290, nominal viscosity 20 cSt and refractive index *n* = 1.40 @ 589 nm) was infiltrated in the cavity to reduce the vibrations.

Transmission spectra were obtained by illuminating the three-cavity resonator under normal incidence with white light from a halogen lamp. The transmitted light was collected through the entrance slit of an imaging spectrograph (PI Acton Spectra Pro 2300i) aligned with the *x-*axis of the top cylinder (Figure 3b) and recorded with a high-sensitivity CCD camera (Andor Newton DU940P–FI). Only a small region of the surface surrounding the contact position was considered, such that *r* ≤ 0.15 mm ≪ *R*, equivalent to a sphere-plane geometry (Figure 1).^23^ A CCD camera image showed the transmitted intensity *I* as a function of the wavelength λ and position *r* (Figure 5). Multibeam interference created resonance peaks in an image, i.e., local maxima of the 2d intensity function *I*(λ, *r*), corresponding to constructive interference. CCD images were recorded at constant time intervals Δ*t* = 0.7 s while decreasing or increasing *d*. Intensity spectra *I*(λ, *r* = 0) at the contact position were extracted from each image and stacked in sequence to create *I*(λ, *t*) spectrograms, where *t* is the elapsed time (Figure 4a).

### Scattering Matrix Method

Transmitted spectra were simulated using a 2 × 2 transfer matrix multiplication method for stratified optical media (also known as scattering matrix method),^15,54^ with refractive indices determined by ellipsometry for Ag and ITO, and nominal value for silicone oil. Details on the method implementation are given in the Supporting Information.

## Supporting Information Available

Details on the transfer matrix multiplication method; Discussion on the Helmholtz–Schrödinger isomorphism under oblique incidence; Calculations of the resonance splitting and hybridization coefficients for the two-cavity and three-cavity resonators (PDF)
Video showing the electric field within a three-cavity resonator as a function of position and wavelength for different thicknesses of the central cavity (AVI)

## Supplementary Material

ph1c01055_si_001.pdf

ph1c01055_si_002.avi
