# Supplementary material for: Understanding and Controlling Mode Hybridization in Multicavity Optical Resonators Using Quantum Theory and the Surface Forces Apparatus
Source: ACS Photonics. 2021 Nov 15;8(12):3517–25. doi: 10.1021/acsphotonics.1c01055 (PMC9446313; doi:10.1021/acsphotonics.1c01055)
Supplement: Supplementary file 1 — ph1c01055_si_001.pdf [file ph1c01055_si_001.pdf]

SUPPORTING INFORMATION FOR

Understanding and controlling mode hybridization in multi-cavity optical resonators using quantum theory and the surface forces apparatus

Bruno Zappone[1,*], *Vincenzo Caligiuri*[1,2,*], *Aniket Patra*[1,3], *Roman Krahne,*[3] *and Antonio De Luca*[1,2]

[1] Consiglio Nazionale delle Ricerche – Istituto di Nanotecnologia (CNR-Nanotec) via P. Bucci 33/C, 87036 Rende (CS), Italy

[2] Università della Calabria – Dipartimento di Fisica, via P. Bucci 31/C, 87036 Rende (CS), Italy

[3] Istituto Italiano di Tecnologia – Optoelectronics Research Line, via Morego 30, 16163 Genova, Italy.

Contains 7 pages (including this) and 1 figure

Transfer matrix multiplication method

Details on the transfer matrix multiplication (TMM) method, also known as the scattering matrix multiplication method can be found in various optics textbook.[1] Here we present a simple implementation for isotropic multilayers under normal incidence. Consider N layers with total thickness L and normal z comprised between an input medium with refractive index n_0 (from $z = -\infty$ to $z = 0$) and an output medium with index n_s (from $z = L$ to $z = +\infty$). In each layer, a monochromatic electric field with time dependence $e^{-i\omega}$ can be written as $E = Fe^{ink(z-a)} + Be^{-ink(z-a)}$, where a is the position of the layer entrance, F is the amplitude of the forward-propagating wave (travelling along z), and B is the amplitude of the backward-propagating wave. The phase of the input field is $a = 0$ at the multilayer entrance, whereas $B = 0$ for the output field. The continuity of the electromagnetic field at the interface between the ith and $i + 1^{th}$ layer can be expressed in matrix form: $S_i P_i \mathbf{A}_i = S_{i+1}\mathbf{A}_{i+1}$, where $\mathbf{A} = (F, B)$ is a 2d vector; $S = (1, 1; n, -n)$ is a 2×2 matrix, $P = (e^{inkl}, 0; 0, e^{-inkl})$ represents wave propagation, and l is the layer thickness. The matrix equation above can rewritten as $S_i\mathbf{A}_i = T_i S_{i+1}\mathbf{A}_{i+1}$ where we have introduced the transfer matrix:

$$T = \begin{bmatrix} \cos kl & -(i/n)\sin kl \\ -in\sin kl & \cos kl \end{bmatrix} \quad \text{(S1)}$$

Using the matrix equations above, the amplitude vector $\mathbf{A}_i$ can be expressed as a function of the vector $\mathbf{A}_j$ of any other layer via a relation $S_i\mathbf{A}_i = M_{ij}S_j\mathbf{A}_j$, with $j > i$, where $M_{ij} = T_i T_{i+1} \ldots T_{j-1}$ is obtained by multiplying the transfer matrices of all layers in between, including the ith layer but excluding the jth layer. In particular, the total reflection and transmission coefficients of the multilayer can be calculated from the matrix $M = T_1 T_2 \ldots\ldots T_N$ using the following formulas:

$$\begin{cases} r_0 = \dfrac{B_0}{F_0} = \dfrac{n_0 M_{11} + n_0 n_s M_{12} - M_{21} - n_s M_{22}}{n_0 M_{11} + n_0 n_s M_{12} + M_{21} + n_s M_{22}} \\ t_0 = \dfrac{F_s}{F_0} = \dfrac{2n_0}{n_0 M_{11} + n_0 n_s M_{12} + M_{21} + n_s M_{22}} \end{cases} \quad \text{(S2)}$$

After determining the output (transmitted) amplitude F_s and reflected amplitude B_0 as a function of the input (incident) amplitude F_0 from Eq. S2, the amplitudes F_i and B_i in any layer can be calculated from Eq. S2 as a function of F_s, after replacing M with $M_{iN} = T_i T_{i+1} \ldots\ldots T_N$, and n_0 with n_i, namely:

$$\begin{cases} t_i = \dfrac{F_s}{F_i} \\ r_i = \dfrac{B_i}{F_i} = t_{is}\dfrac{B_i}{F_s} \end{cases} \quad \text{(S3)}$$

Helmholtz-Schrödinger isomorphism under oblique incidence

In this section we discuss the Helmholtz-Schrödinger isomorphism for a planar multilayer under oblique incidence. The Helmholtz equation can be written as $\nabla^2\psi + \varepsilon(\omega/c)^2\psi = 0$, where ψ is a component of the electric or magnetic field, ε is the dielectric permittivity, ω is the time frequency, and c is the speed of light. We consider layers with normal z made of transparent dielectric material and metal for which ε is respectively positive or negative. To be a solution of the Helmholtz equation, a plane wave $\psi(\mathbf{r}) = A\exp(-i\mathbf{k}\cdot\mathbf{r})$ must satisfy the condition $|\mathbf{k}|^2 = \varepsilon(\omega/c)^2 = k_{||}^2 + k_z^2$, where $\mathbf{r}$ is the position vector, and $\mathbf{k}$ is the wavevector with components $k_{||}$ and k_z in the directions parallel and normal to the layers, respectively. For such wave, the Helmholtz equation becomes $(\partial^2/\partial z^2 + k_z^2)\psi = 0$ and is isomorphic to the steady-state Schrödinger equation $[(\partial^2/\partial z^2) + (2m/\hbar^2)(\mathcal{E} - V)]\psi = 0$. The latter equation describes a hypothetical quantum particle with mass m in a one-dimensional potential V, where $\mathcal{E}$ is the particle energy minus the kinetic energy in the layer plane. The square modulus $k_z^2 = \varepsilon(\omega/c)^2 - k_{||}^2$ can be positive or negative in a dielectric, respectively for $k_{||}^2 < \varepsilon(\omega/c)^2$ and $k_{||}^2 > \varepsilon(\omega/c)^2$. Therefore, the particle energy $\mathcal{E} = V + (\hbar k_z)^2/2m^2$ can be respectively higher or lower than the potential V. On the other hand, k_z^2 is always negative in a metal such as Ag and therefore $\mathcal{E}$ is always smaller than V. Figure 1 shows that a symmetric square-well potential profile $V(z)$ can be created by inserting a dielectric layer (T) either in a non-adsorbing metal (M) or in another dielectric material (T′) with a lower refractive index. In the first case, the potential is maximum in the metal and the particle can only have energy $\mathcal{E} \leq \max(V)$ (Fig. 1(a)). In the metal, the condition $k_z^2 < 0$ corresponds to a non-propagating, exponentially decaying field. Therefore, the particle is in a bound state localized within the well. When the T layer is surrounded by another dielectric T′, two cases are possible. When $k_z^2 > 0$ in both T and T′ materials, for instance under normal incidence, the particle is in a freely propagating state with energy $\mathcal{E} \geq \max(V)$ (Fig. 1(b). On the other hand, when $k_z^2 > 0$ in the T layer, but $k_z^2 < 0$ in the T′ material, the particle is in a bound state with energy $\mathcal{E} \leq \max(V)$, similar to that obtained with th M material. Namely, the case of Fig. 1(c) corresponds to total internal reflection.[1]

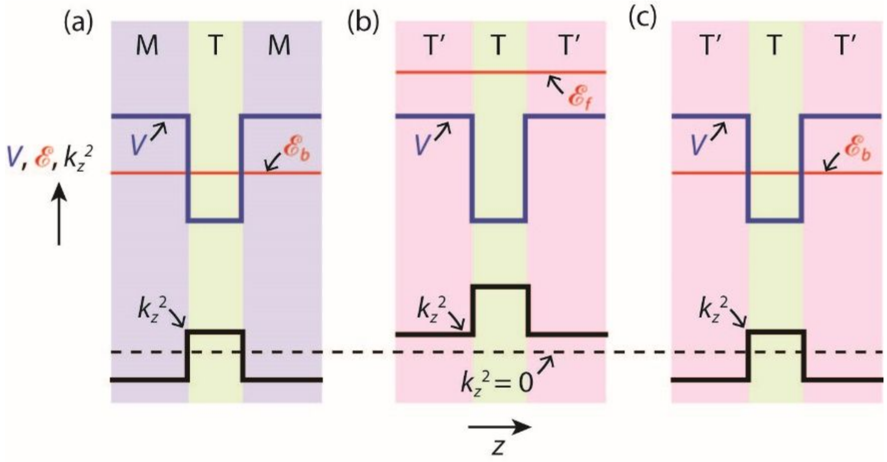

Figure S1. (a) Symmetric square-well potential profile $V(z)$ created by a layer of dielectric material (T) with refractive index n_D surrounded by a non-absorbing metal (M). The particle can only be in a bound state with energy $\mathcal{E}_b \leq \max(V)$. k_z^2 is the square modulus of the normal wavevector. (b) A similar potential is obtained by replacing the metal with another dielectric, T′, having a lower refractive index $n_D' < n_D$. If $k_z^2 > 0$ in the T and T′ materials, the particle is in a freely propagating state with energy $\mathcal{E}_f \geq \max(V)$. (c) Total internal reflection: the particle is in a bound state with energy $\mathcal{E}_b \leq \max(V)$, $k_z^2 > 0$ in T, and $k_z^2 < 0$ in T'.

Although the Helmholtz and Schrodinger are formally identical, the quantum wave function ψ and its first derivative $\partial\psi/\partial z$ are continuous across a potential step,[2] whereas not all components of the electromagnetic field satisfy these requirements across a metal-dielectric interface. To be continuous, the field ψ in the Helmholtz equation must be a component of the electric or magnetic field parallel to interface.[1] The continuity requires that the plane waves have transverse-electric (TE or *s*) polarization or transverse-magnetic (TM or *p*) polarization. However, only TE waves satisfy first-order continuity, as we shall see.

Suppose that a planar metal-dielectric interface located at $z = 0$ is such that the dielectric (T) is located at $z < 0$ and the metal (M) at $z > 0$. The wave function ψ can be written as the sum of an incident and reflected plane wave for $z < 0$, and one transmitted wave for $z > 0$. Although the wavevector square modulus $k^2 = \varepsilon(\omega/c)^2$ and potential V shows a step-like change at $z = 0$ (Fig. S1), the continuity of ψ requires that the parallel wavevector component $\mathbf{k}_{||}$ be conserved across the interface. This requirement entails the usual laws of reflection and refraction (Snell laws) for both optical waves (TE or TM)[1, 3] and quantum particle[2]. For instance, the normal wavevector can take the two values $k_z = \pm|k^2 - k_{||}^2|^{1/2}$ for $z < 0$. The positive and negative values are the normal wavevectors k_{iz} and k_{rz} of the incident and reflected wave, respectively.

We call a_i, a_r, and a_t the amplitude of the incident, reflected, and transmitted wave, respectively. In quantum mechanics, the continuity of ψ and its first derivative $\partial\psi/\partial z$ entails that $a_i + a_r = a_t$ and $k_{iz}(a_i - a_r) = k_{tz}a_t$.[2, 4] In other

terms, the reflection coefficient is $r = a_r/a_i = (k_{iz} - k_{tz})/ (k_{iz} + k_{tz})$ and the transmission coefficient is $t = a_t/a_i = 2k_{iz}/(k_{iz} + k_{tz})$. As demonstrated in various optics textbooks,[1, 3] these relations are satisfied only by plane waves with TE polarization. Therefore, the Helmoltz-Schrödinger isomorphism applies only to TE waves under normal incidence.

Calculations of hybridization coefficients and energy splitting

Trial functions $|\psi\rangle$ for the variational method can be constructed as symmetry-adapted linear combinations (SALCs) of the states $|\alpha\rangle$ of isolated (non-interfering) wells. The SALC coefficients a_α are determined by the variational equation $\partial\mathcal{E}/\partial a_\alpha = 0$, where:

$$\mathcal{E} = \langle\psi|\mathcal{H}\psi\rangle/\langle\psi|\psi\rangle \qquad \text{(S4)}$$

is the average energy of the SALC and $\mathcal{H}$ is the complete Hamiltonian of the interfering wells. The best approximations for the hybridized states $|\psi\rangle$ and their energies $\mathcal{E}$ are obtained for coefficients a_α that are solutions of Eq. (S4).

Symmetric two-cavity resonator

A symmetric two-cavity resonator (MTMTM) can be obtained by bringing into direct contact the outer cavities of a symmetric three-cavity resonator (MTMDMTM), thereby squeezing out the transparent fluid D from the central layer. We call $\mathcal{H}_\alpha = -(\hbar^2/2m)\partial^2/\partial z^2 + V_\alpha$ the Hamiltonian of an isolated well, where $\alpha = l$ or $\alpha = r$ indicate the left and right well, respectively, and V_α is the potential of a single well. The ground (first-order) state $|\alpha_1\rangle$ is the same for both the left and right well has energy $\mathcal{E}_1$. Two SALC trial functions can be constructed: the even state $|g_1\rangle = (|l_1\rangle + |r_1\rangle)/\sqrt{2}$ and the odd state $|u_1\rangle = (|l_1\rangle - |r_1\rangle)/\sqrt{2}$ (Fig. 6a–left side). In the absence of overlap and interference between the wells, these two SALCs have the same energy $\mathcal{E}_1$. The degeneracy, however, is lifted when the wavefunctions of the two wells overlap. The complete Hamiltonian for a double square well is $\mathcal{H}_2 = -(\hbar^2/2m)\partial^2/\partial z^2 + V_l + V_r$, where V_l and V_r are the single-well potentials of the left and right well, respectively. Substituting $|\psi\rangle = |g_1\rangle$ or $|\psi\rangle = |u_1\rangle$ in Eq. (S4), we obtain the average energy:

$$\mathcal{E}_{1g,u} = \mathcal{E}_1 + U(\tau \pm \chi)/(1 \pm \sigma) \qquad \text{(S5)}$$

where $\sigma = \langle l_1|r_1\rangle$ is the overlap integral, $\tau U = \langle l_1|V_r l_1\rangle = \langle r_1|V_l r_1\rangle$, $\chi U = \langle l_1|V_l r_1\rangle = \langle V_r l_1|r_1\rangle$, and $U < 0$ is the depth of the wells. To obtain Eq. (S5), we have used the key property that the Hamiltonian can be decomposed as $\mathcal{H}_2 = \mathcal{H}_\alpha + V_\beta$, where α and β indicate different wells, implying that $\mathcal{H}_2|\alpha_1\rangle = \mathcal{E}_1|\alpha_1\rangle + V_\beta|\alpha_1\rangle$. Note that τ and χ are the square modulus and overlap, respectively, of the left state $|l_1\rangle$ in the right well or, vice versa, of the right

state $|r_1\rangle$ in the left well. When the wavefunctions of the two wells overlap only slightly, we expect $\tau \ll \chi < \sigma \ll 1$. Therefore the average energy of the states $|g_1\rangle$ and $|u_1\rangle$ can be approximated as $\mathcal{E}_{1g,u} \approx \mathcal{E}_1 \pm \chi U$ to the first order in σ in Eq. (S5). Since $\chi U < 0$, the energy levels $\mathcal{E}_{1g}$ and $\mathcal{E}_{1u}$ are respectively below and above the doubly degenerate level $\mathcal{E}_1$.

Symmetric three-cavity resonator

The complete Hamiltonian for a symmetric three-cavity resonator (MTMDMTM) is $\mathcal{H}_3 = -(\hbar^2/2m)\partial^2/\partial z^2 + V_l + V_c + V_r$, where V_c is the potential of the isolated central well. The possible SALCs for the ground state are the odd state $|u_1\rangle$ and the even states $a_c|c_1\rangle + a_g|g_1\rangle$, with coefficients a_c and a_g to be determined from Eq. (S4), leading to a set of two coupled linear equations:

$$\Sigma_{\mu,\nu} a_\mu (H_{\mu\nu} - \mathcal{E}\langle\mu|\nu\rangle) = 0 \qquad \text{(S6).}$$

Here, μ and ν indicate the states $|g_1\rangle$ and $|c_1\rangle$, and $H_{\mu\nu}$ are the matrix elements of the triple-cavity Hamiltonian $\mathcal{H}_3$:

$$H_{\mu\mu} = \langle\mu|\mathcal{H}_3|\mu\rangle = \mathcal{E}_1 + \tau U \qquad \text{(S7)}$$

$$H_{\mu\nu} = \langle\mu|\mathcal{H}_3|\nu\rangle = \mathcal{E}_1\sigma + \chi U \qquad \text{(S8)}$$

where $\sigma = \langle g_1|c_1\rangle$, $\tau U = \langle g_1|V_c g_1\rangle = \langle c_1|(V_l + V_r)c_1\rangle$ and $\chi U = \langle g_1|(V_l + V_r)c_1\rangle = \langle V_c g_1|c_1\rangle$. To derive Eq. (S7-S8), we have used the Hamiltonian decompositions $\mathcal{H}_3 = \mathcal{H}_2 + V_c$ and $\mathcal{H}_3 = \mathcal{H}_c + (V_l + V_r)$. Equation (S6) has a non-trivial solution if the associated determinant is zero, leading to the 2nd order secular equation:

$$\|H_{\mu\nu} - \mathcal{E}\langle\mu|\nu\rangle\|/U = (\tau - \delta)^2 - (\sigma\delta + \chi)^2 = 0 \qquad \text{(S9)}$$

where $\delta = (\mathcal{E} - \mathcal{E}_1)/U$. Equation S9 gives two distinct energy shifts $\delta_\pm = (-\tau \pm \chi)(1 \pm \sigma)$ corresponding to even states with coefficient ratio $(a_g/a_c)_\pm = -(\delta_\pm + \tau)/(\delta_\pm\sigma + \chi)$. Noting again that $\tau \ll \chi < \sigma \ll 1$, the solution of Eq. (S9) to the first order in σ is $\delta_\pm \approx \pm\chi$, that is $\mathcal{E}_\pm = \mathcal{E}_1 \pm \chi U$, with coefficient ratios $(a_g/a_c)_\pm = \pm 1$. Therefore, the two hybrid even states are $|\pm\rangle = (|c_1\rangle \pm |g_1\rangle)/\sqrt{2}$. Since $\chi U < 0$, the $|+\rangle$ state has energy $\mathcal{E}_+ < \mathcal{E}_1$ and a “bonding” character, whereas the $|-\rangle$ state has energy $\mathcal{E}_- > \mathcal{E}_1$ and “anti-bonding” character.

REFERENCES

S1. Fowles, G. R., *Introduction to modern optics*. Dover: New York, USA, 1989.
S2. Messiah, A., *Mécanique quantique*. Dunod: Paris, 1964.
S3. Born, M.; Wolf, E., *Principles of Optics*. Pergamon Press: Oxford, UK, 1980.
S4. Griffiths, J. G., *Introduction to quantum mechanics*. Prentice Hall: New Jersey, USA, 1995.
